# It's Safe to Look: Maternal Touch Affects Infants’ Fear Bias

**DOI:** 10.1111/desc.70039

**Published:** 2025-06-10

**Authors:** Margaret Addabbo, Elena Guida, Victoria Licht, Chiara Turati

**Affiliations:** ^1^ Department of Psychology, CRIdee Università Cattolica del Sacro Cuore Milano Italy; ^2^ Department of Psychology University of Milano‐Bicocca Milano Italy; ^3^ NeuroMI Milan Center for Neuroscience Milano Italy

**Keywords:** eye‐tracker, facial expressions, fear‐bias, infants, mother‐infant interaction, touch

## Abstract

Touch is an extraordinary sensory, communicative, and affective experience that has cascading positive effects on infants’ socio‐emotional development and neurobiological functioning. This study aims to explore whether maternal touch can influence infants’ well‐known attentional biases toward fearful facial expressions. Visual behaviour of 7‐month‐old infants was measured through an eye‐tracker while they were presented with an overlap task in which a central emotional face (happy, neutral, and fearful) was followed by a peripheral distractor. During the task, infants were randomly assigned to two experimental groups. In one group, the mother kept the hand on the infant's lap (Touch group, *N* = 24), while in another group, the mother was present but not in tactile contact with the infant (No‐Touch condition, *N* = 24). Also, the frequency of spontaneous maternal touch was assessed during mother‐infant free‐play interaction. Results showed an overall increase in the proportion of looking times (PTs) toward the fear stimulus compared to the other emotional stimuli. Further, only the group of infants that were in tactile contact with their mothers showed slower disengagement times (DTs) from fearful faces compared to happy and neutral emotions. Finally, in the No‐Touch condition, infants who experienced more regulatory touch (massages and caresses) when interacting with their mother showed increased attention toward threatening (Fearful faces) and ambiguous (Neutral faces) emotional signals. Vice versa, increased frequency with other forms of touch (i.e., dynamic and static) during the interaction was associated with decreased attention toward the negative and neutral facial expressions. Our findings suggest that maternal touch provides a relevant communicative signal to the infant that indicates that it is safe to process fearful stimuli, favoring infants’ knowledge and learning of their social world.

## 1 Introduction

Through maternal touch, infants learn to feel safe and secure in their challenging and sometimes unpredictable environment. Indeed, the experience of touch is extraordinarily rich in sensory, communicative, and affective information and can favorably influence infants’ development and neurobiological functioning (Carozza and Leong 2021; Field 2010; Montirosso and McGlone 2020; Yoshida and Funato 2021). One of the key features that makes the sense of touch “so special” early in human life is its capacity to regulate infants’ emotions and behavior. For instance, when infants are in tactile contact with their mother, they cope better with a stressful event, such as maternal temporary unavailability (i.e., Still‐Face Paradigm) (Feldman, Singer et al. 2010). Indeed, touch reduces infants’ negative reactions and behaviors to stressful situations (Feldman, Singer et al. 2010), induces the suppression of cortisol reactivity (Feldman, Singer et al. 2010; Scott et al. 2022), and promotes oxytocin release, a neuropeptide involved in the formation of the mother‐infant bonding and the development of affiliative behaviors (Feldman, Gordon et al. 2010). Further, affective tactile stimulations regulate infants’ heart rate and respiration (Feldman, Singer et al. 2010; Manzotti et al. 2019; Yoshida et al. 2020; Van Puyvelde et al. 2019), promoting a state of calmness, closeness, and well‐being in the mother‐infant dyad.

**Summary**

- Attention toward fearful, happy, and neutral faces was measured through an eye‐tracker while infants were in tactile contact or not with their mothers.
- An overall increase in looking times was observed toward the fearful faces compared to happy and Neutral expressions.
- Only infants in tactile contact with their mothers showed slower disengagement times from fearful faces.
- When not touched, infants’ attention to emotional faces changed with the frequency of maternal touch during everyday mother‐infant interaction.

The enhancement of neurophysiological and behavioral stability fosters, in turn, infants’ interest and openness in exploring social environments. For example, maternal affective tactile interactions boost 4‐month‐olds' ability to discriminate facial identities (Della Longa et al. 2019), and reduce infants’ avoidance behaviors toward strangers (Tanaka et al. 2021). Recently, it has been shown that affective versus non‐affective mothers’ touch exerts differential effects on infants’ visual behavior toward emotional facial expressions during a preferential‐looking task. Specifically, in frequently touched infants, maternal affective caresses, but not squeezes, reduce infants’ avoidance of angry facial expressions (Addabbo et al. 2021). Beyond infancy, a maternal brief touch on the child's shoulder lowers his/her attention to angry faces (Brummelman et al. 2019). Further, children experiencing frequent maternal touch show increased connectivity in areas that belong to the “social brain” (Brauer et al. 2016). These studies pointed out that parent‐infant tactile interactions can shape how infants and children process and explore their socio‐emotional environment. Based on the above‐reviewed literature, the current study investigated whether maternal touch might have an impact on infants’ processing of emotional faces, and specifically on the well‐established “fear bias” that emerges around the second half of the first year of life (Burris et al. 2019; Peltola et al. 2013).

Infants are capable of discriminating and recognizing emotional cues from faces from very early in life (Addabbo et al. 2018; Geangu et al. 2016). Fearful emotional expressions are particularly attractive for infants. Behavioral evidence is pretty prominent and consistent in showing that such attentional fear bias emerges between 5 and 7 months of age (Heck et al. 2016; Peltola et al. 2013; Leppänen et al. 2018). For example, from 5 months of age, in the presence of a competing stimulus, infants attend more and are slower in disengaging attention from fearful compared to happy or neutral facial expressions (Ahtola et al. 2014; Leppänen et al. 2018; Peltola et al. 2013, 2009, 2015; Heck et al. 2016). Importantly, some key factors, such as infants’ temperament (i.e., negative affect) as well as maternal psychopathology (i.e., anxiety, depressive symptoms, stress), are known to affect and modulate infants’ attentional bias to fearful faces (Forssman et al. 2014; Kataja et al. 2020; Reilly et al. 2022; Vallorani et al. 2021). Increased attention to fearful faces at 7 months is also related to infants’ attachment security at 14 months of life (Peltola et al. 2015). Further, a recent EEG (electroencephalographic) study has found that maternal odor can lower infants’ neural responses to fearful faces (Jessen 2020). Interestingly, many studies that explored infants’ fear bias tested infants while they were in very close body contact with their parent (i.e., on their laps, in the baby carrier) (Ahtola et al. 2014; Heck et al. 2016; Leppänen et al. 2018; Peltola et al. 2015, 2013). Parental tactile contact while attending to emotional visual stimuli may create a safe and secure context that modulates infants’ attention toward fearful emotions.

Here, we conducted an eye‐tracker study to explore whether maternal tactile contact can influence infants’ attention toward fearful facial expressions. We hypothesize that, by signaling safety, maternal touch would increase infants’ attention to fearful faces. As in previous studies (Ahtola et al. 2014; Forssman et al. 2014; Leppänen et al. 2018; Peltola et al. 2015; Heck et al. 2016), we employed the overlap task, in which the infant was first presented with an emotional face (happy, neutral, and fearful) centrally on the screen, followed by a peripheral distractor (i.e., a checkerboard pattern). Crucially, in one group of infants (Touch condition), the mother stood next to the infant throughout the task keeping her hand still on his/her lap, while in a second group of infants (No‐Touch condition), the mother stood next to the infant without having any tactile contact with him/her. We expected infants to show increased attention toward fearful faces when they were in tactile contact with their mother, but not when such body contact was absent. Research on both infants and adults indicates that multiple types of touch can regulate infant behavior. Gentle caressing (Crucianelli and Filippetti 2020) and massage (Mrljak et al. 2022) have well‐documented regulatory effects on behavior and physiological responses early in life. Furthermore, skin‐to‐skin contact is widely recognised for its positive impact on development (Norholt 2020). In adults, holding hands has been shown to convey a sense of comfort and security in couples (Sahi et al. 2021; Ali et al. 2023). Notably, static touch has been successfully employed in previous developmental studies and has proven effective in modulating attentional responses without disrupting children's focus on visual stimuli (Brummelman et al. 2019). In the disengagement task, we opted to use static rather than dynamic touch to minimize interference with infants' visual attention to the emotional faces presented. Static touch offers natural, stable and consistent tactile stimulus, reducing the risk of distractions, or shifts in focus that might accompany dynamic movement. It also minimizes variability in tactile perception, ensuring a uniform experience for the infants. By choosing static touch for the disengagement task, we aimed to ensure that any observed effects on infants’ visual attention were attributable to the presence of touch itself rather than differences in movement, pressure or intensity. This approach allowed us to more clearly investigate how tactile contact influences infants' focus on emotional facial expressions.

Furthermore, we explored whether the natural variation of touch‐related behaviors in the caregiver‐infant dyad has an impact on infants’ attention toward emotional faces. To this aim, following the overlap task, we measured the frequency of maternal spontaneous tactile behaviors during 5 min of free face‐to‐face interaction. Previous studies have shown that the frequency of maternal touch assessed during free mother‐infant interaction or through specific questionnaires (i.e., Parent‐Infant Caregiving Touch Scale, PICTS; Wigley et al. 2023; Addabbo et al. 2021) affects infants’ exploratory behaviors of a novel object, reduce evasive behavior toward a stranger (Tanaka et al. 2021), modulates children's brain connectivity in areas that belong to the “social brain” (Brauer et al. 2016) and infants’ visual preferences toward emotional facial expressions (Addabbo et al. 2021). Importantly, in the disengagement task, we implemented a robust manipulation to determine whether the presence or absence of maternal tactile contact influenced infants' disengagement times from emotional faces. Additionally, the free‐play interaction provided an opportunity to: (1) observe natural variations of touching behaviors within the mother‐infant dyad and (2) explore whether different types of touches had distinct effects on infants' disengagement times from emotional stimuli. Research has shown that certain types of touch, such as slow, gentle strokes, and massages, are particularly effective in regulating infants’ physiology and behavior (Manzotti et al. 2019; Della Longa et al. 2019; Field 2014), especially when compared to other forms of touch like fast strokes or tapping. Notably, slow, gentle strokes are preferentially processed by slow‐conducting unmyelinated nerve fibers known as CT‐fibers, which are believed to encode rewarding and socially meaningful tactile information (McGlone et al. 2014; Morrison et al. 2010). Therefore, we anticipate that among all types of touch, maternal stroking and massages may exert the greatest influence on enhancing infants’ attentional bias toward fearful expressions.

## 2 Methods

### 2.1 Participants

The final sample included 48 healthy full‐term 7‐month‐old infants (24 females; *M*_age_ = 232.2 days; SD = 8.1) recruited from the city center and hinterland of Milan. Fifteen infants were also tested but excluded due to fussiness (*N* = 6) or errors of the eye‐tracker system (*N* = 9). Twenty‐four infants were assigned to the Touch condition (*M* = 234 days, SD = 8.21), and the other half of the sample was assigned to the No‐Touch condition (*M* = 231 days, *SD* = 7.80). No difference in age was found between the two experimental conditions (*t*(46) = 1.48; *p* = 0.14). Further, infants’ temperament (Surgency, Negative Affect, and Effortful control) did not differ in the two experimental conditions (All *p*s > 0.12). Mothers’ anxiety levels (Trait and State) were also comparable in the Touch and No‐Touch conditions (All *p*s > 0.67). A priori power analysis for a repeated‐measures ANOVA with within‐ and between‐subjects factors was conducted (G*Power, as in Cohen (1988) option), using an assumed effect size of *f* = 0.25, correlation among repeated measures of *ρ* = 0.5, and an alpha level of *α* = 0.05. The analysis indicated that a sample size of 44 (22 for each age experimental condition) would be required to achieve a power level of 1−*β* = 0.95.

The protocol was carried out in accordance with the ethical standards of the Declaration of Helsinki (BMJ 1991; 302: 1194) and approved by the Ethical Committee of the University of Milano‐Bicocca (Prot. 746/2023). Parents gave their written informed consent before the testing session began.

### 2.2 Stimuli

The central stimuli consisted of two adult female Caucasian faces selected from the Radboud Faces Database (RaFD) (Langner et al. 2010) expressing happiness, fear, and neutrality with a directed gaze. The original stimuli from the Radboud Faces Database (RaFD) were altered by changing the background color from gray to white. The central face was 10.2° wide and 13° high. The lateralized distractor was a black‐and‐white checkerboard appearing at 13.3° from the center of the screen. The checkerboard was 2.8° wide and 10.3° high.

### 2.3 Procedure

Infants sat on a baby seat in front of the stimulus presentation monitor (24″ screen size, 1920 × 1080 pixel resolution, 60 Hz), at a distance of about 60 cm. The infants’ gaze direction was recorded by a video camera, and the stimulus presentation was designed with E‐Prime 3 (Psychology Software Tools) extension for Tobii Pro Lab. Our eye‐tracker system was a Tobii X3 120 (Tobii Technology AB, Stockholm, Sweden) that samples eye‐tracking data at 120 Hz with a spatial resolution of 0.2°, a drift of 0.1 degrees, and gaze accuracy of around 0.5°.

First, a 5‐point infant calibration procedure was presented to the infant, in which an animated duck paired with an engaging sound appeared in the four corners and center of the screen. The target was presented sequentially: when the infant looked at the target, the experimenter pressed a key to advance to the next one until all the targets were shown to the infant. Following the calibration phase, the overlap task began.

**FIGURE 1:**
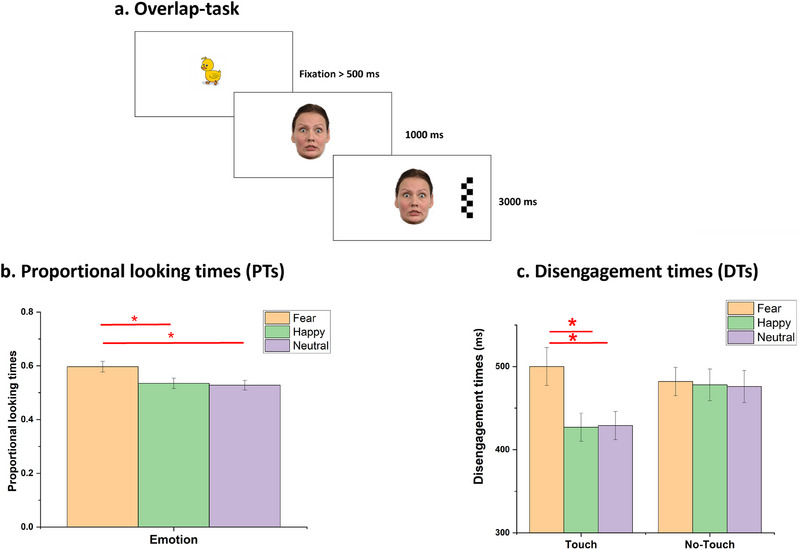
(a) Overlap‐task procedure; (b) PTs toward fearful, happy, and neutral faces; (c) DTs (ms) from the fearful, happy, and neutral faces during the overlap task. Error bars refer to the standard errors of the mean.

When the infant fixated on a flickering yellow duck displayed in the center of the screen for at least 500 ms, the emotional face was presented centrally on the screen and followed by a peripheral distractor—that is, a checkerboard—after 1000 ms (Figure 1a). The distractor acted as an exogenous cue to orient attention to its location. The distractor could appear randomly and equiprobably at the left or the right of the central stimulus. The checkerboard flickered at a rate of 10 Hz for the first 1000 ms and stayed stationary for the remaining 2000 ms (as in Peltola et al. 2009). The emotional face and the checkerboard were shown together on the screen for 3000 ms. The presentation of the six faces (2 identities × 3 emotions) was pseudorandomized, with the only restriction being that the same facial expressions appeared no more than twice consecutively. The trials continued until the baby got fussy or bored and stopped looking at five stimuli in a row. Similarly to Brummelman et al. (2019), infants were randomly assigned to two different experimental conditions: infants in the Touch condition were statically touched on their right leg by their mothers’ right hand, who stood next to them without interacting. The mother kept her hand still on the infant's leg throughout the whole experimental session. In the No‐Touch condition, mothers did not touch infants and stood next to them, on their right, without any form of interaction. In both conditions, the mother stood next to the infant at the same distance and in the same position with their backs turned to the monitor, thus, they did not have visual access to the stimuli presented.

After the eye tracker task, the mother‐child dyads of both conditions were invited to play together for 5 min while sitting in front of each other, without any toys. The mother was sitting on a chair, and the infant was in a high chair, at a distance of 60 cm. The mother‐infant face‐to‐face interaction was recorded by a video camera located on their side. The other side was visible as well thanks to a unidirectional mirror that gave access to the whole full‐face image and scene. The mother was instructed to play freely with her child with no interactive constraints. After the testing session, mothers were asked to fill out the Infant Behavior Questionnaire‐revised very short form (IBQ‐R VSF; Putnam et al. 2014; Montirosso et al. 2011), a parent report to assess infants’ temperament, and the State‐Trait Anxiety Inventory (STAI‐Y; Spielberger, Gorsuch, Lushene, Vagg, & Jacobs, 1983), to measure anxiety levels of mothers.

### 2.4 Eye‐Tracker Data Analysis

Raw data were exported from the Tobii Studio, and interpolation of those periods during which the position of gaze remained within the target area was performed with a maximum temporal gap length of 75 ms. We defined two areas of interest (AOIs), one for the face and one for the peripheral distractor. The shape and dimensions of the AOIs were the same for all emotions and actresses. The face AOI was a square of 10.2° wide and 13° high drawn around the face stimulus. The distractor AOI was a rectangular shape drawn around the checkerboard, 20° in height and 4.7° in width. Individual trials were excluded if the gaze shifts from the face to the peripheral checkerboard were absent, if there were anticipatory looks to the peripheral stimulus location before the stimulus appeared, or within 150 ms of stimulus onset, if infants performed the saccade after 1000 ms from the distractor onset and if the infant was not fixating the face before performing a saccade toward the distractor. These exclusion criteria were used in previous studies that employed the overlap task (i.e., Heck et al. 2016). In line with previous studies (Ahtola et al. 2014), all infants with at least two trials per emotion condition were retained in the analysis. The average number of trials included for the Touch condition was *M* = 8.96 (*SD* = 3.75; range = 2–16) for Fear, *M* = 8.88 (*DS* = 3.05; range = 4–15) for Happy, and *M* = 9.38 (*SD* = 3.83; range = 4–16) for Neutral faces. The average number of trials included in the analysis for the No‐Touch condition was *M* = 9.38 (*SD* = 4.66; range = 2–23) for Fear, *M* = 9.33 (DS = 3.99; range = 3–20) for Happy, and *M* = 9.42 (*SD* = 3.61; range = 2–18) for Neutral faces. A rmANOVA with Emotion (Fear, Happy, Neutral) as a within‐subject factor and Condition (Touch, No‐Touch) as the between‐subject factor revealed no significant main effect or interaction (All *p*s > 0.19).

### 2.5 Behavioral Coding of Touch During Face‐to‐Face Interaction

The 5‐min videos of mother‐infant face‐to‐face interaction were coded offline on a 1‐s basis using ELAN software (Wittenburg et al. 2006).

Two main composite scores were created. The Regulatory‐touch score was the sum of two coding categories (i.e., caresses—slow and gentle strokes on the infant body—and massages—rubbing infants’ body) which are known to possess regulatory effects on infant physiology and behavior (Manzotti et al. 2019; Field 2014); the Other‐touch score was the sum of other touch categories not related to infants’ regulation (i.e., Dynamic touch—repetitive tactile movements, tickling, moving limbs/body, patting—and Static touch—holding limbs, static touch, palmar grasp). Coding categories were based on previous literature (Brzozowska et al. 2021), to capture the full spectrum of possible tactile behaviors occurring during mother‐infant interaction. For each participant, every touch category considered has been calculated as the proportion of each touch category relative to the total of touches during the interaction and then summed to create the two composite scores (Regulatory and Other‐touch). As data of the composite scores were not normally distributed, as assessed by a Kolmogorov–Smirnov test (*p* < 0.05), they were log‐transformed (Ln) to normalize the distribution (Csibra et al. 2016).

During training, coders reviewed discrepancies occurring in the corresponding portion of the video examined. Raters discussed and decided the appropriate type of maternal touch for that particular portion of the video. Coders were blind to the aims and hypotheses of the study. The 25% of videotapes (*N* = 12) were coded by both coders to assess inter‐rater agreement (89%, *k* = 0.81). In case of disagreement between coders, it was solved in conference with a third coder, who has extensive experience in behavioral coding of mother‐child interactions. For every 1‐s segment, coders had to select only one type of touch.

## 3 Questionnaires

The Infant Behavior Questionnaire‐revised very short form (IBQ‐R VSF; Putnam et al. 2014; Montirosso et al. 2011) is a 36‐item scale rated from 0–8 (0 = does not apply, 1 = never, 2 = very rarely, 3 = less than half the time, 4 = about half the time, 5 = more than half the time, 6 = almost always, 7 = always). Items coded as 0 (does not apply) were treated as missing. Items assess three broad temperament dimensions: positive affect/surgency (PAS), negative emotionality (NEG), and orienting and regulatory capacity (ORC). The internal consistency of the IBQ–R VSF scales ranged from 0.70 to 0.92.

The State‐Trait Anxiety Inventory (STAI‐Y; Spielberger, Gorsuch, Lushene, Vagg, & Jacobs, 1983) measured anxiety levels of mothers. It is a self‐assessment questionnaire comprising 40 questions. Items are rated on a 4‐point Likert scale (from “Almost Never” to “Almost Always,” or from “Not at All” to “Very Much So”). Total scores for each part range from 20 to 80. Higher scores indicate more significant anxiety.

## 4 Data Analysis

Two repeated‐measures Analyses of Variance (rmANOVAs) were run. Our dependent variables were proportional looking times durations (PTs) to the face relative to the distractor (i.e., total fixation duration to the face divided by the sum of the total fixation duration to the face and to the distractor) and infants’ disengagement times (DTs), defined as the latency from the checkerboard onset to the time when the infant shifts gaze from the face to the checkerboard (Heck et al. 2016). Independent variables were Emotion (Fear, Happy, Neutral) as the within‐subjects factor and Condition (Touch, No‐Touch) as the between‐subjects factor. To measure the association between touch categories (Regulatory‐touch vs. Other‐touch) in the free‐play session and infants’ DTs and PTs, Pearson's Correlation analyses were used and run separately for the Touch and No‐Touch conditions.

## 5 Results

### 5.1 Proportional Looking Times Durations

The repeated‐measures Analysis of Variance (rmANOVAs) on PTs with Emotion (Fear, Happy, Neutral) as the within‐subjects factor and Condition (Touch, No‐Touch) as the between‐subjects factor revealed a significant main effect of Emotion, *F*(2, 92) = 9.93; *p* < 0.001, *ηp^2^* = 0.178. Within and between‐subjects posthoc comparisons (Tukey corrected) revealed that PTs were greater in response to the Fearful (*M* = 0.60, *SD* = 0.14) compared to the Happy (*M =* 0.53 ms, *SD =* 0.14), *t*(92) = 3.069; *p* = 0.01; *Cohen's d* = 0.442 and Neutral face (*M =* 0.53 ms, *SD =* 0.13), *t*(92) = 4.460; *p* = 0.001*; Cohen's d* = 0.629 (Figure 1b). These results are coherent with previous evidence (Heck et al. 2016), documenting increased visual attention toward fearful faces by 7 months of age.

### 5.2 Disengagement Times

The repeated‐measures Analysis of Variance (rmANOVAs) on DTs with Emotion (Fear, Happy, Neutral) as the within‐subjects factor and Condition (Touch, No‐Touch) as the between‐subjects factor revealed a significant main effect of Emotion, *F*(2, 92) = 4.40; *p* = 0.015, *ηp^2^* = 0.09. However, no comparison reached significance after Tukey correction (All *ps* > 0.05). Interestingly, the rmANOVA also revealed a significant Emotion × Condition interaction, *F*(2, 92) = 3.34; *p* = 0.04, *ηp^2^* = 0.07. Within and between‐subject posthoc comparisons (Tukey corrected) showed that only in the Touch condition, DTs from fearful faces (*M =* 500 ms, *SD =* 111) were slower compared to Happy (*M =* 427 ms, *SD =* 83.3), *t*(92) = 3.026, *p* = 0.04, *Cohen's d* = 0.51, and Neutral faces (*M =* 429 ms, *SD =* 82.7), *t*(92) = 3.138; *p* = 0.03, *Cohen's d* = 0.57. No differences in DTs were found in the No‐Touch condition (Happy: *M =* 478 ms, *SD =* 93.2; Fear: *M =* 482 ms, *SD =* 83.7; Neutral: *M =* 476 ms, *SD =* 94.8; All *p*s > 0.77) (Figure 1c). Results indicate that the tactile contact with the mother affected infants’ attentional disengagement from emotional faces, with infants being less prone to deviate attention from fearful faces when touched.

### 5.3 Behavioral Coding of Maternal Touch and Infants’ PTs and DTs

First, we explored whether the amount of maternal touch coded during the free‐play interaction differed across conditions (Touch vs. No‐Touch). No significant differences were found (All *p*s > 0.16).

Then, we investigated whether, separately for each condition (i.e., Touch and No‐Touch), there was a significant relationship between the frequency of touch (i.e., Regulatory vs. Other‐Touch) measured during the face‐to‐face interaction and infants’ PTs and DTs from fearful, neutral, and happy facial expressions using Pearson's Correlation analyses. The False discovery rate (FDR) of 0.05 adjustment using the Benjamini and Hochberg procedure (Benjamini et al. 2001) was applied to account for multiple comparisons. Four touch scores were missing (*N* = 2 in the Touch and *N* = 2 in the No‐Touch condition) due to technical issues occurring during the mother‐infant interaction.

**FIGURE 2:**
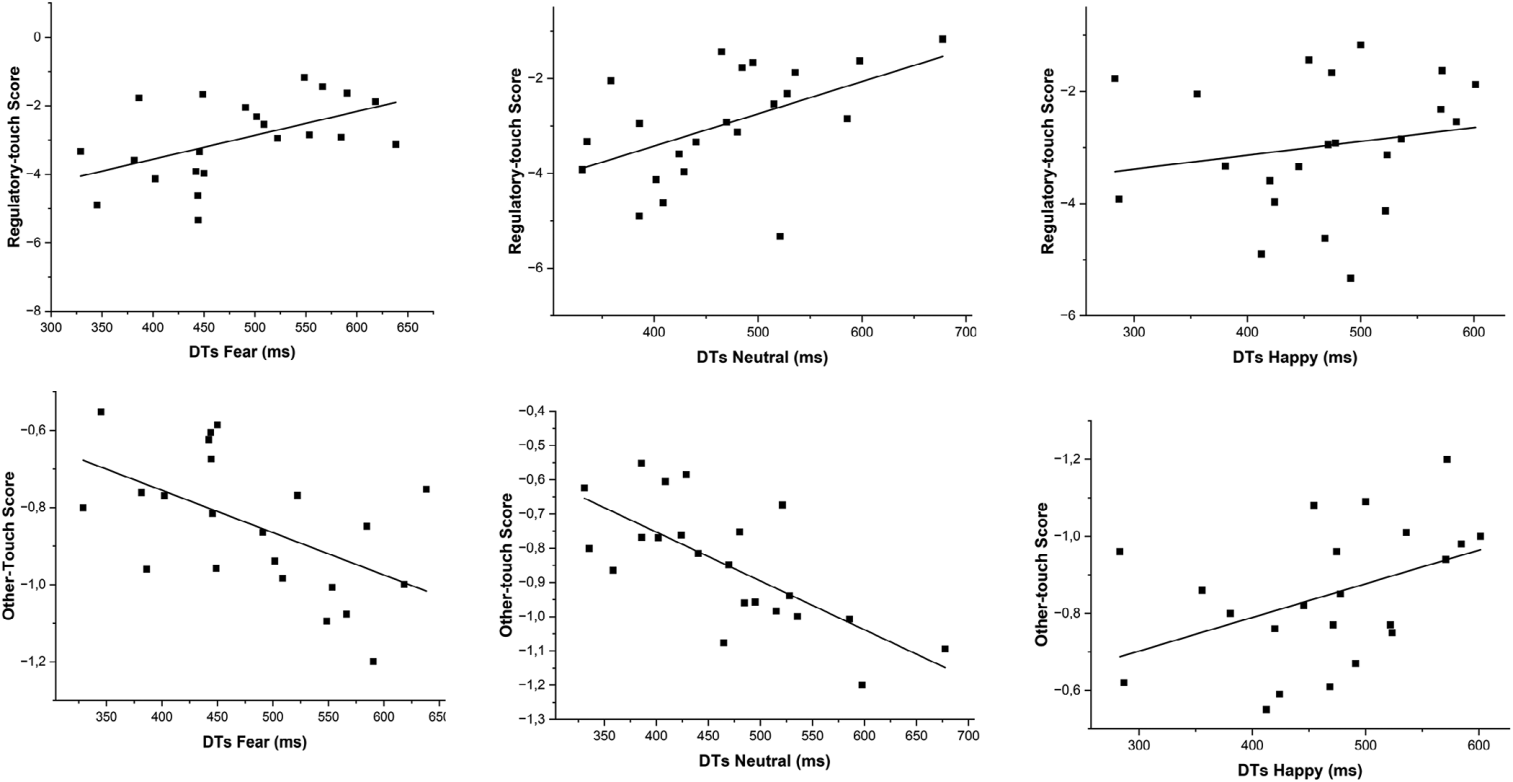
Associations between frequency of regulatory and Other‐touch and infants’ DTs in the No‐Touch condition. Specifically, increased DTs from Neutral and Fearful faces are associated with increased frequency of Regulatory‐touch, while increased DTs from Neutral and Fearful faces are related to decreased frequency of Other‐touch. No association was found between the frequency of touch and DTs from Happy faces.

When considering PTs as the dependent variable, the analysis revealed no significant associations. When considering DTs, the analysis revealed a significant relation only in the No‐Touch condition, between the frequency of Regulatory and Other‐Touch and DTs. In particular, the frequency of Regulatory touch was positively related to DTs from Fearful (*r* = 0.516; *p* = 0.014) and Neutral faces (*r* = 508; *p* = 0.016). Conversely, the frequency of Other‐touch was negatively related to DTs from Fearful (*r *= –0.540; *p* = 0.010) and Neutral touch (*r* = –0.706; *p* < 0.001). Overall, these results indicate that in the absence of concurrent mothers’ touch contact, day‐by‐day touching experience with their mother modulated infants’ looking time, with Regulatory‐touch promoting infants’ visual exploration of face stimuli (Figure 2).

## 6 Discussion

Our findings are in line with previous studies showing that, at 7 months of life, infants show heightened attention to fearful expressions. Indeed, results over the proportion of looking times, reflecting attention‐holding processes, showed an overall greater allocation of infants’ attention toward the fear stimulus compared to the other emotional stimuli. Further, we have shown for the first time that a situation‐dependent social signal, such as maternal tactile contact, is associated with infants’ attention to fearful expressions. In particular, the previously established patterns of infant fear bias over DTs, which is a measure of attention orienting, were observed only in the presence of maternal touch. Indeed, only the infants in tactile contact with their mothers showed slower disengagement times from fearful faces compared to happy and neutral emotions. These results indicate that maternal touch has promoted infants’ attention toward fearful faces, temporarily inhibiting saccades toward competitive distractor stimuli presented in the periphery. Thus, when touched, infants actively prioritized fearful expressions and filtered out other non‐salient stimuli, such as black‐and‐white checkerboards.

It is well known that tactile contact with the mother has regulatory effects on the infant. In the first year of life, infants rely on their parents to regulate their internal states, given that they possess very limited and immature regulatory abilities (Planalp and Braungart‐Rieker 2015). Touch is a very powerful means through which parents can stabilize and balance infants’ behavior and internal physiological states (Carozza and Leong 2021; Field 2010; Yoshida and Funato 2021), which could have important cascading effects on infants’ cognition and attentional resources. In our study, maternal tactile contact might have helped infants down‐regulate the negative arousal elicited by fearful faces, encouraging them to prioritize their attention toward such potentially threatening stimuli. Thus, maternal touch might have prompted an adaptive and appropriate response, providing a relevant communicative signal indicating that it is safe to process negative stimuli. A deeper exploration of fearful faces might boost infants’ learning of emotions that look threatening but that could turn out to be innocuous, favoring infants’ knowledge and learning of their social world. It is worth noting that some evidence has related infants’ negative affect and maternal anxiety to an amplified attention bias toward fearful faces (Forssman et al. 2014; Kataja et al. 2020; Reilly et al. 2022; Vallorani et al. 2021). Conversely, other studies showed that an enhanced fear bias at 7 months of life is related to a secure attachment style at 14 months of life, as measured through the Strange Situation procedure (Peltola et al. 2015) and a heightened attention bias for fearful faces at 8 months is linked to positive outcomes in later socioemotional competencies (Eskola et al. 2023). The results of our study are in line with these latter findings and the idea that the presence of a fear bias should be considered normative and adaptive, as it is a cue reflective of infants’ understanding of the social environment.

Interestingly, infants’ attention toward fearful expressions is not only associated with situation‐dependent tactile contact but also with variations in caregivers’ everyday touching patterns measured during mother‐infant free‐play interactions. In the No‐Touch condition, results show that the more frequently mothers engage in regulatory touch with their infants, the longer the infants’ disengagement times from both fearful and neutral faces. In addition, increased use of Other‐Touch was associated with decreased DTs from Fearful and Neutral emotions. These results indicate that when infants are not experiencing tactile contact during the experimental task (No‐Touch condition), their attention toward social signals is associated with their daily interactive routines with their mother: infants that experience in their daily life with more regulatory touch are more open to explore threatening (Fearful faces) and ambiguous (Neutral faces) emotional signals. Differently, touches in the Other‐touch category seem to be related to a general decrease in attention toward neutral and negative facial expressions. To better interpret such an association, further studies might explore whether touches included in the Other‐touch category tactile stimulations are attuned to infants’ emotions and behavior or are rather inappropriate or intrusive tactile stimulations. Overall, results show that in the absence of contingent physiological and behavioral stability deriving from a situation‐dependent maternal touch, infants’ visual behavior toward Fearful and Neutral expressions might be shaped by variations in the style of caregivers’ touching patterns. Differently, in the touch condition, no association was found between mother‐infant touching patterns and infants’ attention to emotional faces. This result could suggest that contingent maternal tactile contact overshadows the influence of habitual tactile experience within the mother‐infant dyad. The effect of both habitual and contingent experiences with maternal touch on infants' visual behavior was also found in a recent study investigating 7‐month‐olds' visual preferences for angry versus happy emotions (Addabbo et al. 2021). However, it should be highlighted that our study's moderate statistical power and small sample size may impact the results' generalizability to the broader population.

Results from the disengagement task highlight that maternal contact alone serves as a powerful signal of safety, being associated with infants' gaze behavior toward emotional cues in unfamiliar situations. In addition, findings from the free‐play interactions revealed that different types of maternal touch could have distinct effects on infants' disengagement times from emotional stimuli. One may claim that participation in the disengagement task prior to the free‐play session may have influenced the mother's subsequent tactile behaviors with her infant. However, it is important to highlight that the free‐play session took place in the same way as all mothers were asked to interact with their infants as they normally would. Future studies may nonetheless further investigate the influence of spontaneous tactile mother‐infant interactions on infants’ processing of social stimuli. Studying mothers and infants in their natural environments would allow researchers to capture tactile behaviors reflecting real‐life interactions, preserving the richness and authenticity of their everyday dynamics. Future research should also investigate the effects of various types of touch (e.g., strokes, massages, still contact) to determine which most effectively fosters a sense of safety and calmness in infants, consequently influencing their disengagement times from negative emotions.

Our findings, consistent with previous research in infancy (Addabbo et al. 2021), indicate that maternal touch influences infants’ attention to emotions by encouraging visual exploration of threatening faces. This contrasts with the findings of Brummelman et al. (2019), who reported that maternal touch reduces vigilance toward angry faces in school‐age children and has no effect during adolescence. Overall, this pattern of results highlights significant developmental changes in how children process social signals. In infancy, maternal touch encourages infants to explore those social signals that are still not entirely known by infants, such as fearful emotional faces. However, as children grow and become capable of efficiently decoding those negative social cues, maternal touch may serve a different function, potentially reducing vigilance toward faces expressing negative emotions. Then, the effects of maternal touch decline in late childhood, indicating that as children transition into adolescence, parental touch may lose its role as a signal of safety (Brummelman et al. 2019). During adolescence, the regulatory role of maternal touch appears to diminish. Adolescents increasingly seek independence from their parents and may instead turn to significant others for tactile comfort and connection (Brummelman et al. 2019). Indeed, evidence shows that parents may exert their greatest influence on amygdala activity—a subcortical structure that mediates emotional learning and attention/arousal for emotionally relevant stimuli—during the early stages before adolescence (Tottenham 2015). Notably, Jessen (2020) found that maternal odor reduces 7‐month‐olds’ neural responses to fearful faces over the Nc, indexing attention allocation. Thus, this study seems to contrast with our findings. However, the relation between eye‐tracker and neural measures is not as straightforward as it seems. A recent study (Xie et al. 2021) did not find an association between behavioral measures of attention disengagement and neural responses to emotional faces in infancy. Such a relation emerged only later in development, at 36 months of life. At this age, the N290 component showed a positive association with children's fear and anger bias scores, while the P400 and Nc components demonstrated a negative association. This suggests that children who pay less attention to negative emotions (as shown by the Nc, indexing attention allocation) may need a longer time to fully process faces, as reflected by looking times toward negative emotions. Future research could explore the effects of maternal touch by integrating ERP and eye‐tracking measures for a more detailed understanding of its effect on the behavioral and neural levels.

There is something that makes fearful faces so attention‐grabbing early in life, and that distinguishes this emotion not only from other innocuous emotions (happiness) but from other threatening expressions as well, such as anger. Some scholars suggested that such attentional bias is guided by the threatening value of fearful faces (e.g., Leppänen and Nelson 2012). According to these authors, it might be adaptive for infants to pay attention to fearful faces, especially during the second half of the first year of life, when they begin to be autonomous in locomotion, as they signal a potential danger for the infant. An alternative explanation of the fear attentional bias refers to the infants’ poorer experience with fearful faces, which can be considered unfamiliar stimuli, given that infants are rarely exposed to such expressions compared to other emotions, such as happiness and anger (Campos et al. 2000). Our results do not disentangle between these two alternative interpretations; however, they do show that the previously established attentional biases toward fearful faces in infancy emerged exclusively when the child was in contact with their mother. Maternal touch might encourage infants to explore potentially threatening, fearful facial expressions, offering them new opportunities to learn from their social environment.

Understanding the environmental situational factors associated with infants’ processing of emotional cues opens a window on the pathways to support infants’ emerging socio‐emotional skills. Our results shed light on the unique role that parental touch has in infants’ attention toward salient social stimuli in their environment, delineating the nurturing role of touch early in human development.

## Ethics Statement

All data were collected in accordance with the ethical principles of the Declaration of Helsinki (BMJ 1991; 302: 1194). This research received approval by the Ethical Committee of the University of Milano‐Bicocca (Prot. 746/2023).

## Conflicts of Interest

The authors declare no conflicts of interest.

## Data Availability

Data are available from the OSF: https://osf.io/y62p8/
